# The use of complementary and alternative medicine (CAM) in children: a telephone-based survey in Korea

**DOI:** 10.1186/1472-6882-12-46

**Published:** 2012-04-20

**Authors:** Jung-Ha Kim, Chung-Mo Nam, Moo-Young Kim, Duk-Chul Lee

**Affiliations:** 1 Department of Family Medicine, Chung-Ang University Medical Center, Seoul, South Korea; 2 Department of Preventive Medicine, Yonsei University College of Medicine, Seoul, South Korea; 3 Department of Biostatistics, Yonsei University College of Medicine, Seoul, South Korea; 4 Department of Family Medicine, Severance Hospital, Yonsei University College of Medicine, Seoul, South Korea; 5 Department of Family Medicine, Severance Hospital, Yonsei University, College of Medicine, 250 Seongsanno, Seodaemun-gu, 120-752, Seoul, South Korea

## Abstract

**Background:**

The purpose of this study was to estimate the prevalence and patterns of CAM use in Korean children via a telephone based survey. We also investigated parent satisfaction, a proxy for their child, with CAM therapy and determined the factors affecting satisfaction with CAM use.

**Methods:**

This study used a landline telephone-based survey to examine a random sample representative of Korean children, aged 0 to 18 years. We assigned and surveyed 2,000 subjects according to age group, gender, and geographical distributions by proportionate quota and systematic sampling of children throughout Korea in 2010. A household of 1,184 with a 18.6% response rate was projected to yield 2,077 completed data. We performed statistical analyses using sampling weight.

**Results:**

The prevalence of CAM use was 65.3% for the Korean children in our sample population. The most commonly used CAM category was natural products (89.3%). More than half of CAM user’s parents reported satisfaction with their therapies (52.7%), but only 29.1% among them had consulted a Western trained doctor regarding the CAM therapies used. Doctor visits were associated with lower satisfaction with CAM use but not with consultation rate with a doctor.

**Conclusions:**

Our study suggests that CAM is widely used among children in Korea. Medical doctors should actively discuss the use of CAM therapies with their patients and provide information on the safety and efficacy of diverse CAM modalities to guide the choices of CAM users.

## Background

The types and classifications of complementary and alternative medicine (CAM) differ by country because CAM modalities are individually related to a country’s conventional health system and medical curriculum [1]. Furthermore, the evidence base for CAM remains relatively weak, although recent years have seen increased scientific rigor for the study of CAM modalities. Despite the lack of scientific evidence, the use of CAM is increasing worldwide [2-4]. It has been reported that 27–74.8% of adults use CAM [5-7], and there is also an increasing tendency toward its use in children worldwide [6-8].

In the United States (US), the 2007 National Health Interview Survey (NHIS) reported that 11.8% of children surveyed had used CAM therapy in the previous 12 months [6]. In East Asia, the use of CAM is influenced by both culture and local heath care systems; therefore, a separate study of CAM use among children in East Asia would be especially useful. Thus far, the only national surveys of CAM use have been conducted in the US.

In surveys regarding CAM use, satisfaction levels after CAM use are generally high, even higher than satisfaction with conventional primary care [9]. Satisfaction is a multi-dimensional concept in the health care system [10]. Satisfaction with healthcare is not associated with the effectiveness of a treatment alone because satisfaction depends on an individual’s attitudinal response to the consumer’s judgment which is formed by personal characteristics, such as values, beliefs, experiences, personality, health status, and sociodemographics [10]. To improve the quality of healthcare service, in order to meet the diverse and individual needs of patients, it is necessary to identify the factors that affect CAM user satisfaction.

Hence, the purpose of this study was to estimate the prevalence and patterns of CAM use in Korean children via a telephone based survey. We also investigated parent satisfaction with CAM therapy as a proxy for their child and identified factors affecting satisfaction with CAM use.

## Methods

### Participants and sampling

We surveyed the parents or caregivers of 2,077 non-institutionalized Korean children between the ages of 0 and 18 years. To ensure that the surveyed population was a nationally representative sample, we applied proportionate quota and systematic sampling (Additional file 1) methods to the distribution of children in Korea and stratified a total of 2,000 children by age group (0–2, 3–6, 7–12, 13–15, and 16–18 years), gender, and geographical area (25 regions) [11]. We administered telephone-based surveys to the parents or caregivers between July 12 and July 21, 2010 using a list-assisted random-digit dialing method and a landline telephone directory. We made random phone calls to households in each region to ask if they had children under the age of 18. Up to three attempts (from 9 am to 11 am on weekdays, after 9 pm on weekdays, and on weekends) were made to reach each of the telephone numbers. The study was complete when we reached the quota for each age group.

**Figure 1:**
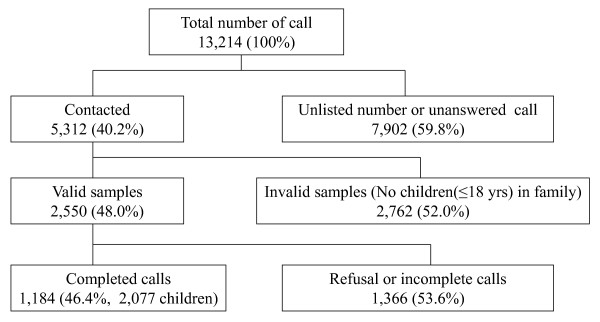
Overall response rate to the landline telephone survey.

Among 13,214 phone numbers dialed, 59.8% were either unlisted or non-responsive numbers. Of the 5,312 responding households, 52.0% did not have a family member within the target age group (children under age 18) and thus were excluded from our study. Of the remaining 2,550 samples, 1,366 households were excluded due to refusal to participate or because the calls could not be completed. Ultimately, we acquired complete data for 2,077 children from 1,184 households (Figure 1). The response rate was 18.6%, according to the definition of The American Association for Public Opinion Research (the number of complete interviews with reporting unit divided by the number of eligible reporting unit in the sample: [1,184/2,550 + (0.48*7902)]*100) [12]. In our survey, 427 households (36.1%) had one child, 636 households (53.7%) had two children, 109 households (9.2%) had three children, 9 households (0.8%) had four children, and 3 households (0.3%) had five children. The demographic characteristics of the children’s mother (*n* = 980), as a proxy for the child, surveyed are shown in Additional file 2.

The Institutional Review Board of Severance Hospital, Yonsei University College of Medicine approved the survey. We obtained verbal informed consent for participation from a parent or caregiver, and the interview, including consent, was recorded.

### Survey

Our questionnaire comprised questions pertaining to CAM obtained from the 2007 National Health Interview Survey [13] performed by the Centers for Disease Control and Prevention (CDC), which were suitably modified for our survey population (Additional file 3). We modified the type of question and did not ask a separate question for each CAM therapy. Religious healing such as praying for one’s own health or having others pray for one’s health and vitamin/mineral supplements were included in the definition of CAM use. Also, the reference period for the use of natural products was longer (12 months) in our survey than in the US survey (30 days). Five medical doctors, one pediatrician and four family physicians with extensive knowledge of CAM and one epidemiologist developed the survey used in our study.

We collected information related to sociodemographics, health status, and CAM use. We employed the definition of CAM used by the National Center for CAM (NCCAM), which defines CAM as a group of diverse medical and health care systems, practices, and products that are not generally considered part of conventional medicine [13]. Sociodemographic data included gender, age, geographical location, feeding method (breast milk or formula) of infants, and number of siblings for each child; age, relationship to the child, and educational level of the parent or caregiver; and family income. Health-related questions included the perceived health status of the child, specific self-reported illnesses or health problems, and the number of doctor visits in the past 12 months. Questions related to CAM covered its use and type in the past 12 months, the reasons for its use, its out-of-pocket costs for the child and adult family members, perceived effectiveness of the CAM modality, satisfaction with treatment, any adverse effects, and whether a Western-trained physician was consulted concerning its use.

### Statistical analysis

We categorized types of CAM into the following four groups according to the NCCAM classification system: natural products, mind-body medicine, manipulative and body-based practices, and other practices [6]. Natural products included dietary supplements such as non-prescription vitamins and minerals, other non-vitamin and non-mineral supplements, herbs including Korean Oriental Medicine (KOM), aromatherapy, phytoncide therapy (forest bathing to breathe in phytoncides emitted by plants and trees in order to improve health), detoxification regimens, and diet-based therapies such as a macrobiotic diet. Mind-body medicine included prayer limited to praying for health reasons, Taekwondo, Japanese fencing (a system of mental and physical training practiced using bamboo swords), hapkido (a Korean martial art), yoga, deep breathing, and music therapy. Manipulative and body-based practices included chiropractic manipulation, massage, and osteopathy. Other CAM practices included acupuncture, moxibustion, hand acupuncture, and several other practices for health reasons.

The sociodemographic categories that we examined included the subject’s age (≤3, 4–6, 7–12, and ≥13 years), residential region (metropolitan or rural), monthly family income (<1, 1–1.9, 2–2.9, 3–3.9, 4–4.9, and ≥5 x10^6^KRW), caregiver age (20–29, 30–39, 40–49, 50–59, and ≥60 years), caregiver education level (less than high school diploma, high school diploma, college diploma, and postgraduate coursework), and caregiver relationship to the child.

In order to increase the power of this analysis, a child’s perceived health status was classified as “good,” including excellent or good, or “poor,” including fair, poor, or very poor; and satisfaction was classified as “satisfied,” including completely satisfied or somewhat satisfied, or “dissatisfied,” including somewhat dissatisfied or completely dissatisfied. The top two categories and the bottom two categories of caregiver age, the top two categories of caregiver education level, and the bottom two categories of monthly family income were each merged into a single group because the number of respondents in the lowermost and uppermost categories was too small for meaningful statistical analysis.

We conducted descriptive analyses of the responses to the survey questionnaire. Data are presented as mean ± standard deviation, number (weight %), or median (25^th^–75^th^ percentile). We performed statistical analyses using sampling weight, and the results are expressed as a weight percentage (weight %). We compared baseline characteristics between CAM users and non-CAM users using *t*-tests for continuous variables and the chi-square test for categorical variables (*n* = 2,077). Generalized estimating equations (GEE) for ordinal data was used to identify factors associated with satisfaction with CAM to account for multiple responses (*n* = 1,365). We used multivariate logistic regression models to examine the factors associated with consultation with a doctor concerning CAM use (*n* = 1,365). We used the SAS system for Windows V8 (SAS Institute, Cary, NC, USA) for all statistical analyses.

## Results

Among the children, 65.3% had used CAM therapies in the past 12 months, for an average of 1.68 modalities per child, and 887 (74.9%) households had used CAM for at least one child. Approximately half of the children were male (52.5%) and resided in a metropolitan area (50.0%). The mean age of the children was 10.10 ± 5.19 years. In terms of health, 1648 (82.4%) subjects reported a good health status and 446 (22.3%) reported medical problems. In addition, 1730 (86.5%) subjects had visited a doctor during the previous 12 months, and the median number of doctor visits was 4.7 (2.5–7.0). The majority of adults surveyed were the children’s mothers (80.3%). Most caregivers were in their third (41.4%) or fourth (48.5%) decade of life, and 93.4% had at least a high school diploma. Over half of the adult family members (52.5%) used CAM therapies. Table 1 summarizes the characteristics of the children. We found significant differences between CAM users and non-CAM users in the following parameters: region of residence, perceived health status of the child, self-reported illness, number of doctor visits, surveyed caregiver’s relationship to the child, caregiver age and educational level, monthly family income, and CAM use by adult family members (all *p* < 0.01).

**Table 1:** **Characteristics of the study participants according to the use of complementary and alternative medicine (CAM) (*n*** **= 2,077)**

Characteristic	CAM user (N = 1,365)	Non-CAM user (N = 712)	Standard Errors	*P*-value^a^
Gender				0.11
Male	704 (64.71)	384 (35.29)	1.45	
Female	661 (66.84)	328 (33.16)	1.50	
Region of residence				<0.01
Metropolitan	691 (68.96)	311 (31.04)	1.46	
Others	674 (62.70)	401 (37.30)	1.47	
Age	10.13 ± 4.83	10.22 ± 5.42		0.54
Siblings				0.66
None	274 (64.17)	153 (35.83)	2.32	
1 sibling	850 (66.77)	423 (33.23)	1.32	
≥2 siblings	241 (63.93)	136 (36.07)	2.47	
Perceived health status				<0.01
Good	1080 (63.19)	629 (36.81)	1.17	
Poor	285 (77.45)	83 (22.55)	2.18	
Self-reported illness				<0.01
Yes	383 (80.80)	91 (19.20)	1.81	
No	982 (61.26)	621 (38.74)	1.22	
Doctor visit				<0.01
Yes	1224 (68.46)	564 (31.54)	1.10	
No	141 (48.79)	148 (51.21)	2.94	
Number of doctor visits	5.5 (2.9–7.8)	3.2 (1.8–5.6)		<0.01
Relationship to subject				<0.01
Father	207 (58.15)	149 (41.85)	2.61	
Mother	1139 (68.29)	529 (31.71)	1.14	
Grandparent	19 (35.85)	34 (64.15)	6.59	
Caregiver age (years)				<0.01
20–29	20 (57.14)	15 (42.86)	8.36	
30–39	590 (69.33)	261 (30.67)	1.58	
40–49	671 (65.66)	351 (34.34)	1.49	
50–59	57 (53.27)	50 (46.73)	4.82	
≥60	19 (35.85)	34 (64.15)	6.59	
Caregiver educational level				<0.01
Less than high school diploma	41 (35.04)	76 (64.96)	4.41	
High school diploma	606 (65.44)	320 (34.56)	1.56	
College or university diploma	664 (69.38)	293 (30.62)	1.49	
Above postgraduate course	47 (74.60)	16 (25.40)	5.48	
Family income (10^6^ KRW/month)				<0.01
<1	14 (31.82)	30 (68.18)	7.02	
1–1.9	83 (50.30)	82 (49.70)	3.89	
2–2.9	283 (58.35)	202 (41.65)	2.24	
3–3.9	456 (69.51)	200 (30.49)	1.80	
4–4.9	251 (73.18)	92 (26.82)	2.39	
≥5	278 (72.40)	106 (27.60)	2.28	
CAM use by adult family members				<0.01
Yes	808 (81.62)	182 (18.38)	1.23	
No	557 (51.24)	530 (48.76)	1.52	
CAM use expenditures of adult family members^b^	118.55 ± 1049.36	63.27 ± 740.76		0.47

Data are expressed as a number (%), median (25^th^–75^th^ percentile), or mean ± standard deviation.

^a^*P*-values were calculated by *t*-test or *χ*^2^-test.

^b^ Unit: 10,000 KRW/12 months.

Natural products (89.3%) were the most commonly used modality (Table 2). Multi-vitamins and minerals (30.9%) placed first in the dietary supplements category and ginseng (16.2%) came second, followed by probiotics (12.1%), KOM (11.5%), omega-3 (2.0%), vitamin C (1.2%), and others (3.2%). The reasons for using these therapies included increased physical fitness (66.4%), prevention of specific diseases or symptoms (20.1%), treatment of specific diseases or symptoms (5.6%), improved concentration (2.4%), weight loss (0.2%), and others (7.9%). Adverse effects were rare (0.27%), and no serious adverse effects were reported.

**Table 2:** **Frequencies of child complementary and alternative medicine (CAM) use by type (*n*** **= 2,190**^**a**^**)**

CAM categories and specific modalities	Used during the past 12 months	
	Number	weight% (Standard error)
Natural products		
Dietary supplements	1686	77 (0.90)
Diet-based therapies	261	11.9 (0.69)
Other^b^	8	0.3 (0.12)
Mind-body medicine		
Prayer for health reasons	182	8.3 (0.59)
Taekwondo, Japanese fencing, Hapkido	135	6.2 (0.52)
Yoga	41	1.9 (0.29)
Other^c^	5	0.2 (0.10)
Manipulative and body-based practices		
Massage	8	0.4 (0.13)
Other CAM practices		
Acupuncture or moxibustion	21	1.0 (0.21)
Spa for health reasons	14	0.6 (0.17)
Others^d^	10	0.5 (0.15)

^a^ Total number of modalities among the 1,365 children who used CAM.

^b^ Aromatherapy, phytoncide therapy, and detoxification.

^c^ Deep breathing and music therapy.

^d^ Traditional healer, healing touch, and energy therapy.

We found that more than half of the parents of CAM users reported satisfaction (52.7%) with their child’s therapies. Using GEE for ordinal data to evaluate satisfaction with CAM use, parents of boys were found to be more satisfied than parents of girls (OR: 1.261, 95% CI: 1.070–1.486). Although the CAM utilization rate of children younger than 3 years of age was the lowest (49.9%), satisfaction with CAM therapies was the highest for these children’s parents (OR: 1.920, 95% CI: 1.286–2.866). Satisfaction was lower for parents of children using natural products (OR: 0.256, 95% CI: 0.192–0.340) and other CAM modalities (OR: 0.196, 95% CI: 0.085–0.455) than those using mind-body medicine. Doctor visits were associated with lower satisfaction with CAM use (OR: 0.695, 95% CI: 0.525–0.921) (Table 3).

**Table 3:** **Factors associated with satisfaction after complementary and alternative medicine (CAM) use by children (*n*** **= 1,365)**

Factor	Odds ratio (95% CI)	*P*-value
CAM therapy groups		
Natural products	0.256 (0.192–0.340)	<0.01
Manipulative and body-based practices	3.429 (0.631–18.629)	0.15
Other CAM practices	0.196 (0.085–0.455)	<0.01
Mind-body medicine	Reference	
Gender		
Male	1.261 (1.070–1.486)	<0.01
Female	Reference	
Region of residence		
Metropolitan	0.848 (0.719–1.000)	0.04
Others	Reference	
Age (years)		
≤ 3	1.920 (1.286–2.866)	<0.01
4–6	1.319 (1.022–1.702)	0.02
7–12	1.236 (1.002–1.526)	0.02
≥13	Reference	
Perceived health status^a^		
Good	1.428 (0.954–2.138)	0.08
Poor	Reference	
Self-reported illness		
Yes	0.896 (0.737–1.088)	0.15
No	Reference	
Doctor visit		
Yes	0.695 (0.525–0.921)	0.01
No	Reference	
Relationship to subject		
Father	0.813 (0.311–2.131)	0.97
Mother	1.095 (0.421–2.849)	0.58
Grandparent	Reference	
Caregiver age (years)		
<40	1.356 (0.852–2.157)	0.18
40–49	1.266 (0.827–1.939)	0.24
≥50	Reference	
Caregiver educational level		
Less than high school diploma	1.027 (0.546–1.929)	0.93
High school diploma	1.016 (0.851–1.213)	0.73
Above college or university diploma	Reference	
Family income (10^6^ KRW/month)		
<2	0.886 (0.582–1.350)	0.62
2–2.9	0.932 (0.711–1.221)	0.6
3–3.9	0.903 (0.716–1.138)	0.28
4–4.9	1.100 (0.849–1.426)	0.52
>5	Reference	
CAM use by adult family members		
Yes	1.244 (1.044–1.483)	0.01
No	Reference	

Odd ratios have been adjusted for CAM group, gender, region of residence, age, perceived health status, self-reported specific illness, doctor visit, relationship to subject, caregiver age, caregiver educational level, family income, and CAM use by adult family members for a GEE for ordinal data.

^a^ The perceived health status of children was classified as “good,” including excellent or good, or “poor,” including fair, poor, or very poor.

We found that 29.1% of those surveyed had consulted a Western-trained physician concerning using CAM therapies in children. Perception of a good health status (OR: 0.514, 95% CI: 0.275–0.959) and self-reported illnesses (OR: 1.645, 95% CI: 1.242–2.177) were factors related to consultation with a physician (Table 4).

**Table 4:** **Factors associated with consultation with a doctor among complementary and alternative medicine (CAM) users (*n*** **= 1,365)**

Factor	Odds ratio (95% CI)	*P*-value
Gender		
Male	1.109 (0.861–1.429)	0.42
Female	Reference	
Region of residence		
Metropolitan	1.239 (0.956–1.608)	0.11
Others	Reference	
Age (years)		
≤ 3	1.679 (0.928–3.037)	0.09
4–6	1.611 (1.074–2.415)	0.02
7–12	1.158 (0.831–1.613)	0.39
≥13	Reference	
Perceived health status^a^		
Good	0.514 (0.275–0.959)	0.04
Poor	Reference	
Self-reported illness		
Yes	1.645 (1.242–2.177)	<0.01
No	Reference	
Doctor visit		
Yes	0.835 (0.536–1.301)	0.43
No	Reference	
Relationship to subject		
Father	0.964 (0.258–3.603)	0.96
Mother	1.398 (0.374–5.222)	0.62
Grandparent	Reference	
Caregiver age (years)		
<40	1.020 (0.497–2.093)	0.96
40–49	0.794 (0.404–1.560)	0.50
≥50	Reference	
Caregiver educational level		
Less than high school diploma	0.503 (0.182–1.395)	0.19
High school diploma	0.819 (0.623–1.077)	0.15
Above college or university diploma	Reference	
Family income (10^6^ KRW/month)		
<2	0.796 (0.429–1.477)	0.47
2–2.9	0.865 (0.573–1.306)	0.49
3–3.9	0.742 (0.515–1.068)	0.11
4–4.9	0.923 (0.618–1.379)	0.69
>5	Reference	
CAM use by adult family members		
Yes	1.203 (0.919–1.574)	0.18
No	Reference	

Odds ratios and *p*-values have been adjusted for gender, region of residence, age, perceived health status, self-reported specific illness, doctor visit, relationship to subject, caregiver age, caregiver educational level, family income, and CAM use by adult family members in the multivariate logistic regression analysis.

^a^ The perceived health status of children was classified as “good,” including excellent or good, or “poor,” including fair, poor, or very poor.

## Discussion

Between 2009 and 2010, we found the prevalence of CAM use in surveyed Korean children to be 65.3%, which is much higher than the prevalence reported in Western countries. The most commonly used CAM category was natural products (89.3%) even though satisfaction with natural products was relatively low. Visiting a doctor was the only factor related to satisfaction with CAM use among the following three health-related factors of perceived health status, self-reported illness, and doctor visit. Only 29.1% of CAM users’ parents had consulted a Western-trained physician regarding the CAM therapies their children used.

It has been reported that the worldwide prevalence of CAM use is 29–58% in pediatric outpatients [14-18] and 24.5–84.5% in pediatric oncology patients [19]. The prevalence of CAM use in children reported in a national survey of the general population (11.8%) was much lower than that in pediatric patients in the US [20]. In the current study, we found a high prevalence of CAM use in South Korean children. Similar to US reports [6], we corrected the prevalence for vitamin and mineral supplements and religious healing and still found a notable prevalence of 50.7%. The high prevalence may be related to the popular use of traditional Korean Oriental Medicine (KOM). In contrast to Western health care models, traditional KOM is practiced alongside conventional medicine in Korea, which may contribute to the higher use of CAM in Korean children.

There was no difference in the overall prevalence of CAM use between boys and girls. However, the prevalence and type of CAM use did differ by age and gender. CAM use was higher in female adolescents over 13 years of age (66.40%) than in male adolescents (58.64%) of the same age. In particular, the use of mind-body medicine was significantly higher in female adolescents (39.20% versus 25.91%). Korean adolescents study and compete vigorously for entrance to high-ranking universities, which may contribute to the high rates of serious physical or psychological problems in this age group [21]. Female adolescents have a higher prevalence of stress-related disorders, such as depression and irritable bowel syndrome [22,23]. Moreover, the rates of suicidal ideation and suicide attempts have also been shown to be higher among Korean female adolescents in comparison to males [24]. The increased use of mind-body medicine in female adolescents may be related to these social circumstances.

South Korean children used natural products much more frequently than other CAM modalities (89.3%) according to our study. In contrast, although the US study did not include the use of multi-vitamins and minerals in the natural products category, a study of US children found that many different modalities were widely used among CAM users, including natural products (3.9%), chiropractic or osteopathic manipulation (2.8%), deep breathing exercises (2.2%), yoga (2.1%), and homeopathy (1.3%) [6]. Another difference between CAM modalities used in South Korea and those used in the US was the use of homeopathy in the US, especially among children. Highly dilute homeopathic remedies are probably considered safe although there is conflicting evidence regarding the efficacy of homeopathy [25]. However, in Korea, homeopathy is not accepted as a legal form of medical care.

The strongest factor associated with CAM use in children was the use of CAM by adult family members. We found that children whose adult family members used CAM were more than three times as likely to use CAM compared to children whose parents did not use CAM. Contrary to previous US national surveys [6,26,27], the prevalence of CAM use in the Korean children that we surveyed was not related to gender, region of residence, parental educational level, or family income. However, we confirmed the prevalence of CAM use among Korean children to be related to age, the presence of an illnesses or symptom, and medical treatment by a doctor [6].

Approximately half of the parents of CAM users (49.4%) reported that their children’s CAM therapies were effective. The rate of parent satisfaction (52.7%) with CAM was higher than the rate of parent perception of effectiveness of CAM use. This finding was in accordance with previous results that satisfaction was higher for CAM use even though patient-reported symptom relief was significantly poorer among CAM users in comparison to conventional care patients [9], although there was a difference in the respondents between previous studies and this study, i.e. CAM users themselves versus a proxy, respectively. Although the use of natural products was most prevalent, the reported effectiveness and satisfaction of natural products users were significantly lower than those of users of mind-body medicine. Unexpectedly, satisfaction with other CAM practices, including the KOM techniques of acupuncture and moxibustion, was lowest. In previous studies, satisfaction and attitudes toward acupuncture among Western-trained medical doctors as well as patients were favorable [28,29]. Furthermore, KOM belongs to the Korean conventional healthcare system and is covered by national health insurance. Further analysis is necessary to investigate KOM separately from the other CAM practices.

Additionally, the satisfaction of parents of CAM users was higher for children younger than 13 years compared to those older 13 years. Among infants and toddlers, decisions regarding CAM modality, as well as CAM usage, are completely dependent on the will of the parents. Moreover, the practical use of CAM, such as administration according to the recommended dosage and method, can be more strictly applied in infants and toddlers than in adolescents because of parent involvement. Therefore, parents of infants and toddlers may be more satisfied with CAM use. Further studies may be necessary to directly evaluate adolescents’ satisfaction with CAM use.

Interestingly, visiting a doctor was linked to lower satisfaction with CAM use, although it was one of the factors positively associated with CAM use. However, there was no difference between the doctor visit group and the no visit group in the rate of consultation with a doctor concerning CAM use. Parent satisfaction rate and physician consultation concerning CAM use were higher for children between 4 and 6 years of age than for children over 13 years of age, regardless of whether or not the children were treated by a medical doctor. The relationship between low parental satisfaction with their children’s CAM use and doctor visits may be due to the existence of more serious diseases or conditions in these children. Finally, provision of proper information by a doctor may improve parental satisfaction with CAM use. However, many people do not discuss their CAM use with a doctor, and doctors often do not ask about their patients’ CAM use [30-32]. The American Academy of Pediatrics has distributed a poster, developed by the Complementary and Alternative Research and Education (CARE) program, that encourages open communication between pediatricians and families concerning complementary health care practices [33]. Additionally, NCCAM has been conducting an educational campaign, “Time to Talk,” which encourages patients and their health care providers to openly discuss CAM use [34]. For such discussions to be successful, significant knowledge of and reasonable attitudes toward CAM are explicitly necessary among medical doctors, especially given the high rate of CAM usage in Korea. In fact, a recent study reported that there is a growing desire to learn more about CAM among Korean medical doctors [29].

This study had some limitations. First of all, our results are limited by a low response rate (18.6%). Accordingly, the generalizability of these results to the general population of South Korea may not be proper. The high number of unlisted or non-responsive telephone numbers in this study may have contributed to the main reason for the low response rate, suggesting that more than three attempts to connect a call is necessary to reduce the bias. Further, racial and social characteristics of Koreans may be another cause of the low response rate. Although we could not correct for the limitation of the low response rate in this study, stratification with 125 cells with age and geographic area according to a national representative data may have made up for the weak points of our results. Next, evaluation about the representativeness of our respondents was difficult. Respondents in this study were also heterogeneous including mother, father, and grandparents. We could not find any national statistics for mothers, as the respondents that were mainly surveyed, that had children under the age of 18 in Korea. To examine the representativeness of our data, we had to use the data of married women under the age of 49 with children from the Korea National Statistics Office. Thus, the two data may be somewhat different. There was a noticeable distinction between national and our data in educational level and residential district. Compared to the national demographic data, our mother respondents were of a somewhat higher educational level and lived more so outside of metropolitan areas. However, educational level may have been exaggerated by the respondents due to the telephone survey. Proxies other than the child’s mother may have responded as a result of many working mothers in metropolitan areas. That is also another factor that prevents the generalization of our conclusions to the general population of children in South Korea. Nonetheless, this study was the first national survey to attempt to estimate the prevalence of CAM use and explored the factors associated with its use in Korean children. Third, we asked an open-ended question concerning CAM use and did not ask questions on the specific types of CAM therapies used in Korea. Therefore, our definition of CAM was broad and may have overestimated the true prevalence of its use. Fourth, there is the potential for recall bias because subjects responded to survey questions from memory. Fifth, there is a possibility of sampling bias and response bias when using a landline telephone survey. Telephone surveys are limited to households with landline phones, and 53.6% of people who answered the telephone declined our survey. Finally, we could not directly include consultation with a doctor in the multivariate logistic regression model to explore factors associated with satisfaction because we did not question the participants about consultation with a doctor for each CAM modality used.

In conclusion, CAM use was prevalent among the children of Korea in our sample population. Korean medical doctors should actively discuss the use of CAM therapies with their patients and provide information on the safety and efficacy of diverse CAM modalities in order to help guide the choices of CAM users.

## Conclusions

The prevalence and patterns of CAM use in children may be significantly different between Korea and Western countries. Satisfaction with CAM may be related not only to doctor visits, but also to consultation with a doctor concerning CAM therapies.

## Abbreviations

CAM: Complementary and alternative medicine; NHIS: National Health Interview Survey; CDC: Centers for Disease Control and Prevention; NCCAM: National Center for Complementary and Alternative Medicine; KOM: Korean oriental medicine; KRW: Korean won; OR: Odds ratio; CI: Confidence interval; KNHANES: Korea Health and Nutrition Examination Survey; SD: Standard deviation.

## Competing interests

The authors declare that they have no competing interests. The authors alone are responsible for the content and writing of this article.

## Authors’ contributions

All authors have contributed significantly and are responsible for this research. Jung-Ha Kim, MD, contributed through the acquisition and interpretation of data and drafting of the article. Chung-Mo Nam, PhD, performed statistical analyses and data interpretation. Moo-Young Kim, MD, contributed by interpreting the data and critically revising the article to ensure the inclusion of important content. Duk-Chul Lee, MD, PhD, contributed to the conception and design of the study, interpretation of data, and revision of the article. All authors are in agreement with the content of the manuscript. All authors read and approved the final manuscript.

## Pre-publication history

The pre-publication history for this paper can be accessed here:

http://www.biomedcentral.com/1472-6882/12/46/prepub

## Supplementary Material

Additional file 1 The number of target and collected samples. Click here for file

Additional file 2 Demographic characteristics of the children’s mother as proxy respondents. Click here for file

Additional file 3 Questionnaire concerning your child’s CAM use. Click here for file
