# Supplementary material for: The use of complementary and alternative medicine (CAM) in children: a telephone-based survey in Korea
Source: BMC Complement Altern Med. 2012 Apr 20;12:46. doi: 10.1186/1472-6882-12-46 (PMC3461443; doi:10.1186/1472-6882-12-46)
Supplement: Additional file 1 — The number of target and collected samples. [file 1472-6882-12-46-S1.doc]

**Additional file 1.** The number of target and collected samples

| Age(yrs) | 0∼2 | | | 3∼6 | | | 7∼12 | | | 13∼15 | | | 16∼18 | | | Total target | Total collection |
| --- | --- | --- | --- | --- | --- | --- | --- | --- | --- | --- | --- | --- | --- | --- | --- | --- | --- |
|  | T^a^ | C^b^ | W^c^ | T | C | W | T | C | W | T | C | W | T | C | W |
| **Sum (No)** | **248** | **191** | **1.3** | **338** | **369** | **0.9** | **648** | **726** | **0.9** | **379** | **424** | **0.9** | **387** | **367** | **1.1** | **2,000** | **2,077** |
| Seoul City | 46 | 35 | 1.3 | 62 | 63 | 1 | 120 | 123 | 1 | 70 | 71 | 1 | 71 | 67 | 1.1 | 369 | 359 |
| Busan City | 15 | 15 | 1 | 21 | 24 | 0.9 | 41 | 40 | 1 | 24 | 27 | 0.9 | 24 | 20 | 1.2 | 125 | 126 |
| Daegu City | 12 | 8 | 1.6 | 17 | 20 | 0.9 | 33 | 36 | 0.9 | 19 | 18 | 1.1 | 19 | 17 | 1.1 | 101 | 99 |
| Incheon City | 14 | 11 | 1.3 | 19 | 20 | 1 | 37 | 48 | 0.8 | 21 | 31 | 0.7 | 22 | 26 | 0.8 | 113 | 136 |
| Gwangju City | 8 | 8 | 1.1 | 12 | 17 | 0.7 | 22 | 32 | 0.7 | 13 | 15 | 0.9 | 13 | 14 | 0.9 | 68 | 86 |
| Daejeon City | 8 | 8 | 1 | 11 | 12 | 0.9 | 22 | 24 | 0.9 | 13 | 15 | 0.8 | 13 | 14 | 0.9 | 67 | 73 |
| Ulsan City | 6 | 4 | 1.5 | 8 | 6 | 1.4 | 16 | 21 | 0.8 | 9 | 13 | 0.7 | 10 | 9 | 1.1 | 50 | 53 |
| Gyeonggi-do |  |  |  |  |  |  |  |  |  |  |  |  |  |  |  |  |  |
| Urban | 53 | 40 | 1.3 | 72 | 75 | 1 | 138 | 143 | 1 | 81 | 81 | 1 | 83 | 76 | 1.1 | 427 | 415 |
| Rural | 11 | 7 | 1.6 | 15 | 19 | 0.8 | 29 | 33 | 0.9 | 17 | 17 | 1 | 17 | 16 | 1.1 | 90 | 92 |
| Gangwon-do |  |  |  |  |  |  |  |  |  |  |  |  |  |  |  |  |  |
| Urban | 4 | 4 | 1.1 | 6 | 6 | 1 | 11 | 13 | 0.9 | 7 | 8 | 0.8 | 7 | 7 | 1 | 35 | 38 |
| Rural | 3 | 2 | 1.4 | 4 | 4 | 0.9 | 7 | 8 | 0.9 | 4 | 4 | 1 | 4 | 4 | 1.1 | 22 | 22 |
| Chungcheongbuk-do |  |  |  |  |  |  |  |  |  |  |  |  |  |  |  |  |  |
| Urban | 5 | 4 | 1.2 | 6 | 7 | 0.9 | 12 | 12 | 1 | 7 | 7 | 1 | 7 | 6 | 1.2 | 38 | 36 |
| Rural | 3 | 2 | 1.4 | 4 | 4 | 1 | 8 | 8 | 0.9 | 4 | 5 | 0.9 | 4 | 3 | 1.5 | 23 | 22 |
| Chungcheongnam-do |  |  |  |  |  |  |  |  |  |  |  |  |  |  |  |  |  |
| Urban | 4 | 4 | 0.9 | 5 | 4 | 1.2 | 10 | 13 | 0.7 | 6 | 6 | 0.9 | 6 | 5 | 1.1 | 29 | 32 |
| Rural | 6 | 3 | 2 | 8 | 8 | 1 | 16 | 16 | 1 | 9 | 9 | 1 | 10 | 9 | 1.1 | 49 | 45 |
| Jeollabuk-do |  |  |  |  |  |  |  |  |  |  |  |  |  |  |  |  |  |
| Urban | 6 | 2 | 3 | 8 | 6 | 1.4 | 16 | 16 | 1 | 9 | 9 | 1 | 9 | 5 | 1.9 | 48 | 38 |
| Rural | 3 | 3 | 0.9 | 4 | 3 | 1.2 | 7 | 6 | 1.1 | 4 | 3 | 1.3 | 4 | 2 | 2 | 21 | 17 |
| Jeollanam-do |  |  |  |  |  |  |  |  |  |  |  |  |  |  |  |  |  |
| Urban | 4 | 5 | 0.7 | 5 | 4 | 1.3 | 10 | 14 | 0.7 | 6 | 9 | 0.6 | 6 | 7 | 0.8 | 30 | 39 |
| Rural | 5 | 4 | 1.2 | 7 | 16 | 0.4 | 13 | 26 | 0.5 | 7 | 12 | 0.6 | 8 | 11 | 0.7 | 39 | 69 |
| Gyeongsangbuk-do |  |  |  |  |  |  |  |  |  |  |  |  |  |  |  |  |  |
| Urban | 6 | 4 | 1.6 | 9 | 10 | 0.9 | 16 | 18 | 0.9 | 10 | 11 | 0.9 | 10 | 13 | 0.8 | 51 | 56 |
| Rural | 6 | 6 | 1 | 8 | 11 | 0.7 | 15 | 18 | 0.8 | 9 | 13 | 0.7 | 9 | 11 | 0.8 | 46 | 59 |
| Gyeongsangnam-do |  |  |  |  |  |  |  |  |  |  |  |  |  |  |  |  |  |
| Urban | 10 | 4 | 2.5 | 14 | 13 | 1 | 26 | 28 | 0.9 | 15 | 20 | 0.8 | 15 | 10 | 1.5 | 80 | 75 |
| Rural | 7 | 4 | 1.7 | 9 | 11 | 0.8 | 17 | 22 | 0.8 | 10 | 13 | 0.8 | 10 | 9 | 1.2 | 54 | 59 |
| Jeju-do |  |  |  |  |  |  |  |  |  |  |  |  |  |  |  |  |  |
| Urban | 2 | 2 | 1.2 | 3 | 4 | 0.8 | 6 | 6 | 1 | 4 | 5 | 0.7 | 4 | 4 | 0.9 | 19 | 21 |
| Rural | 1 | 2 | 0.4 | 1 | 2 | 0.6 | 2 | 2 | 1.1 | 1 | 2 | 0.6 | 1 | 2 | 0.7 | 7 | 10 |

^a^Target, ^b^Collection, ^c^weight.
