# Supplementary material for: The use of complementary and alternative medicine (CAM) in children: a telephone-based survey in Korea
Source: BMC Complement Altern Med. 2012 Apr 20;12:46. doi: 10.1186/1472-6882-12-46 (PMC3461443; doi:10.1186/1472-6882-12-46)
Supplement: Additional file 2 — Demographic characteristics of the children’s mother as proxy respondents. [file 1472-6882-12-46-S2.doc]

**Additional file 2.** Demographic characteristics of the children’s mother as proxy respondents (*n*=980)

| Characteristic | National Data^a^ | Telephone-Surveyed Data | *P^b^*-value |
| --- | --- | --- | --- |
| Age (years) |  |  | 0.32 |
| 20-29 | 7.2(0.01) | 2.6(0.51) |  |
| 30-39 | 43.3(0.02) | 44.5(1.59) |  |
| 40-49 | 49.4(0.02) | 53.0(1.59) |  |
| Educational level |  |  | <0.01 |
| Less than high school diploma | 15.5(0.01) | 2.7(0.52) |  |
| High school diploma | 52.6(0.02) | 46.2(1.59) |  |
| College or university diploma | 29.7(0.02) | 49.0(1.60) |  |
| Above postgraduate course | 2.2(0.01) | 2.1(0.46) |  |
| Region of residence |  |  | <0.01 |
| Metropolitan | 84.3(0.01) | 49.9(1.60) |  |
| Others | 15.7(0.01) | 50.1(1.60) |  |
| Number of children |  |  | 0.62 |
| A child | 23.8(0.02) | 29.7(1.46) |  |
| 2 children | 63.4(0.02) | 57.3(1.58) |  |
| ≥3 children | 12.9(0.01) | 13.0(1.07) |  |

Data are expressed as a percentage (standard error).

^a^ Korea National Statistics Office (2005).

^b^ *P*-values were calculated by χ^2^-test.
