# Supplementary material for: The use of complementary and alternative medicine (CAM) in children: a telephone-based survey in Korea
Source: BMC Complement Altern Med. 2012 Apr 20;12:46. doi: 10.1186/1472-6882-12-46 (PMC3461443; doi:10.1186/1472-6882-12-46)
Supplement: Additional file 3 — Questionnaire concerning your child’s CAM use. [file 1472-6882-12-46-S3.doc]

**Additional file 3**. Questionnaire concerning your child’s CAM use

1. Do you have any member in your family who were born after August 1991?

__________ members

( Questionnaire was discontinued if no family member fit this age group)

2. What is your ______child’s gender?

3. What is your ______ child’s date of birth?

(If you have children born after August 2007, go to question 3-1 )

3-1. (If your child is under 35 months) What kind of milk are you feeding your child?

① Breast Milk (Go to question 4)

② Formula Milk (Go to question 3-2)

③ Breast milk & formula milk (Go to question 3-2)

④ Not feeding any form of milk (Go to question 4)

3-2. If you are giving your child breast milk and formula milk, do you add anything with the milk? (For example, Clostridiums, probiotics, etc.)

4. Do you think your _______child is in good health?

① excellent ② good ③ fair ④ poor ⑤ very poor

5. Does your _______child have any pre-existing disease or health issues?

① Yes (Please specify )

② No

6. Has your______ child recently visited a hospital?

① Yes (__times / year) ② No

7. Has your ______child undergone any CAM in the recent 12 months?

(Please specify any supplementation, therapy or practices done other than prescribed medication or treatment)

① Yes ② No (if not applicable, please go to question 15)

8. What kind of CAM is your ______ child using? Please specify.

| CAM 1 | CAM2 | CAM3 | CAM4 | CAM5 |
| --- | --- | --- | --- | --- |
|  |  |  |  |  |

※ Please check for each CAM used (9~12).

|  | **9.** Reason for CAM use | 10. Effect of CAM use | 11. Satisfaction after CAM use | 12. Adverse events |
| --- | --- | --- | --- | --- |
| CAM1 | 1. Treatment/Symptom relief 2. Prevention of disease/symptoms 3. Health promotion 4. To boost concentration 5. Weight Reduction 6. Others | 1. Excellent 2. Good 3. Fair 4. Poor 5. Very poor | ① completely satisfied  ② somewhat satisfied  ③ neither satisfied nor  dissatisfied  ④ somewhat dissatisfied  ⑤ completely satisfied | 1. No 2. Yes   (Please specify) |
| CAM2 |  |  |  |  |
| CAM3 |  |  |  |  |
| CAM4 |  |  |  |  |
| CAM5 |  |  |  |  |

13. How much did you spend on the CAM stated above? (_________KRW/ month)

① Under KRW 10,000 ② KRW 10,000~50,000

③ KRW 50,000~100,000 ④ Over KRW 100,000

14. Have you ever consulted a doctor regarding CAM use?

① Yes (Go to question 15) ② No (Go to question 14-1)

14-1. What is your reason for not consulting a doctor concerning CAM use?

① Fear of upsetting the doctor

② Fear of the doctor asking you to not use CAM

③ Felt no need to consult a doctor

④ Didn’t have a chance to consult a doctor because you haven’t visited a hospital lately.

⑤ Others ( )

II. Sociodemographics of parent respondents

15. You are your child’s 1) Father 2) Mother

16. When were you born? Year ____ Month_________

17. Final Education?

1) Junior High School

2) High School

3) University

4) Above graduate School

18. How much do you earn? (_________KRW/ month)

19. Has any other family member used CAM during this last year?

① Yes (Go to 19-1) ② No

19-1. How much have your adult family members (excluding children and adolescents) spent on CAM ? Average ________KRW/ month
